# Mechanical and energetic determinants of impaired gait following stroke: segmental work and pendular energy transduction during treadmill walking

**DOI:** 10.1242/bio.051581

**Published:** 2020-07-21

**Authors:** Gustavo Balbinot, Clarissa Pedrini Schuch, Henrique Bianchi Oliveira, Leonardo A. Peyré-Tartaruga

**Affiliations:** 1 Exercise Research Laboratory, Universidade Federal do Rio Grande do Sul, 750 Felizardo Street, Porto Alegre, 90690-200, RS, Brazil; 2 KITE - Toronto Rehabilitation Institute - University Health Network, Lyndhurst Centre, 520 Sutherland Drive, Toronto, M4G 3V9, ON, Canada

**Keywords:** Stroke, Gait, Oxygen consumption, Mechanics, Energetics, Rehabilitation

## Abstract

Systems biology postulates the balance between energy production and conservation in optimizing locomotion. Here, we analyzed how mechanical energy production and conservation influenced metabolic energy expenditure in stroke survivors during treadmill walking at different speeds. We used the body center of mass (BCoM) and segmental center of mass to calculate mechanical energy production: external and each segment's mechanical work (W_seg_). We also estimated energy conservation by applying the pendular transduction framework (i.e. energy transduction within the step; R_int_). Energy conservation was likely optimized by the paretic lower-limb acting as a rigid shaft while the non-paretic limb pushed the BCoM forward at the slower walking speed. W_seg_ production was characterized by greater movements between the limbs and body, a compensatory strategy used mainly by the non-paretic limbs. Overall, W_seg_ production following a stroke was characterized by non-paretic upper-limb compensation, but also by an exaggerated lift of the paretic leg. This study also highlights how post-stroke subjects may perform a more economic gait while walking on a treadmill at preferred walking speeds. Complex neural adaptations optimize energy production and conservation at the systems level, and may fundament new insights onto post-stroke neurorehabilitation.

This article has and associated First Person interview with the first author of the paper.

## Abstract (precis)

**Summary:** Walking after a stroke may be energetically consuming. Here, we show how compensations and asymmetries may contribute to increasing the amount of work needed to walk following a stroke.

## INTRODUCTION

Focal cerebral ischemia remains the third leading cause of death in industrialized countries, and about 50% to 75% of all stroke survivors have residual motor disabilities including walking impairments (Dirnagl et al., 1999; Sacco et al., 2013; Tiozzo et al., 2015). People living with stroke-related disabilities are often unable to walk for long distances without drastically increasing metabolic demands (cost of transport, C), leading to muscular fatigue (Donelan et al., 2002; Peterson et al., 2010; Tiozzo et al., 2015). Given that walking abilities are fundamental to socialization and performing activities of daily living (ADL), this metabolic burden reduces the quality of life of stroke survivors.

Human walking can be modeled as an inverted pendulum. In this model, the body center of mass (BCoM), potential energy (Ep) and kinetic energy (Ek) are in a continuous exchange to minimize mechanical work and energy production (Gordon et al., 2009; Saibene and Minetti, 2003; Tesio and Rota, 2019). When this optimal walking mechanic is changed, abnormal trajectories of the BCoM and body segments may lead to greater mechanical work production or reduced energy exchange, ultimately influencing the energy cost of locomotion (Massaad et al., 2007). Neural adaptations to overcome the post-stroke gait demands a twofold increase in metabolic energy consumption during walking (Detrembleur et al., 2003; Stoquart et al., 2012). This greater metabolic cost of the hemiparetic gait is mainly associated with increasing external mechanical work (W_ext_) performed by the non-paretic limb to lift the BCoM (Mahon et al., 2015; Stoquart et al., 2012). Interestingly, recent studies have shown that faster and more symmetric walking patterns are energetically advantageous for chronic stroke survivors (Awad et al., 2015, 2016) and healthy subjects (Selgrade et al., 2017). To the best of our knowledge, insights on how the chronic post-stroke gait mechanics adapt to walking at different treadmill speeds are lacking. This can provide information on how stroke survivors may overcome this metabolic burden, by using different walking speeds on a treadmill. Moreover, imbalance between paretic and non-paretic limbs play a major role on body symmetry, affecting the mechanics and energetics of walking (Mahon et al., 2015; Neptune et al., 2004; Stoquart et al., 2012), which are also underexplored in previous studies (Detrembleur et al., 2003; Massaad et al., 2010; Stoquart et al., 2012). Understanding how mechanical work is produced between segments may unveil nuances of chronic neural adaptations following a stroke, at the system level. The bulk of these findings may indicate specific targets for rehabilitation interventions or robotics aiming at increasing independence to walk and to perform ADLs.

**Fig. 1.**
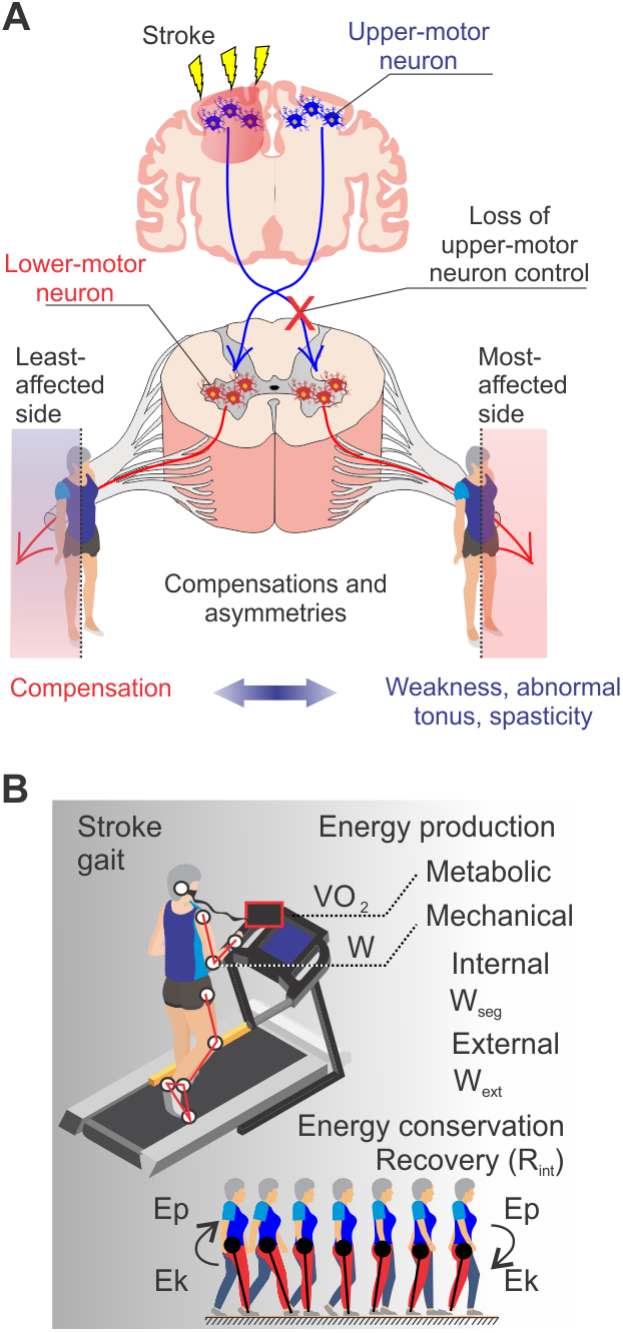
**Changes in mechanical work and cost of transport following chronic neural adaptations to loss of upper-motor neuron control.** (A) Stroke causes loss of upper-motor neuron control over voluntary movements, leading to weakness, abnormal tonus and spasticity at the most affected side and movement compensations at the least affected side (Balbinot and Schuch, 2019). (B) The balance between energy production and conservation maintains a functional post-stroke gait. Ep, BCoM potential energy; Ek, BCoM kinetic energy.

The purpose of this study was to understand the adaptations in optimizing treadmill walking at different speeds following a stroke, for this we described walking mechanics using mechanical energy production and conservation frameworks (Cavagna et al., 2002; Saibene and Minetti, 2003) (Fig. 1). We also conducted a correlational analysis to explore the relationship between the metabolic and mechanical variables. We hypothesized that the increase of C may be accounted for by specific contributions of paretic and non-paretic hemibodies in producing and recovering mechanical energy. We provided insights for further work on how to improve rehabilitation strategies focusing on walking speed and compensations. This may guide rehabilitation programs in improving socialization, independence and ADLs of stroke survivors or further insights on the use of robotics in increasing post-stroke walking efficiency.

## RESULTS

### Mechanical work and cost of transport

**Fig. 2.**
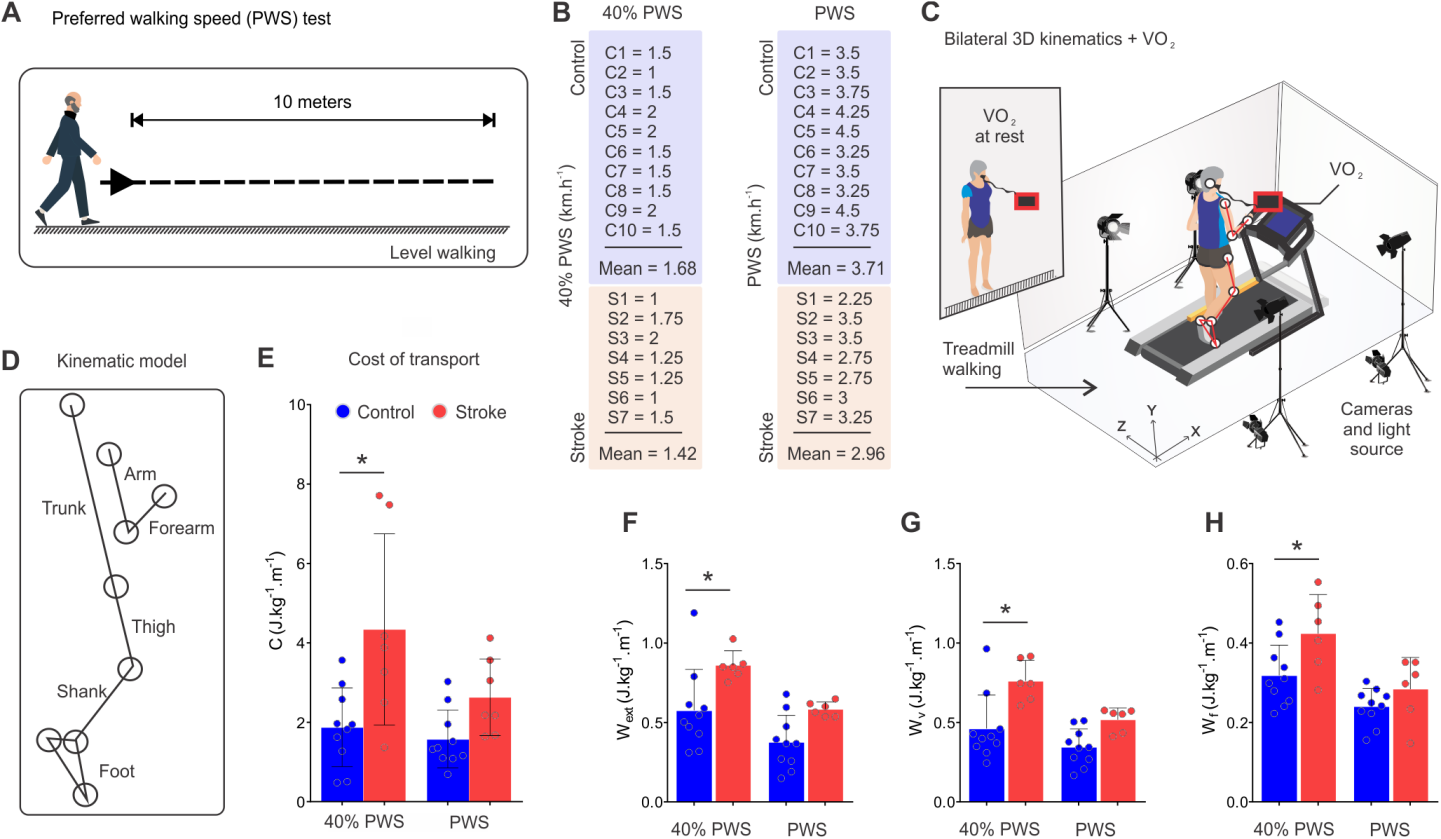
**Metabolic and mechanical energy production was greater for stroke subjects.** (A) A familiarization session was conducted to determine the PWS during level walking. (B) Based on the PWS, subjects were asked to walk on a treadmill at two or three different speeds below the PWS and one or two speeds slightly above the PWS. (C) During each walking speed, bilateral three-dimensional (3D) kinematics and VO_2_ consumption were acquired following 3 min of walking acclimation. (D) The kinematic model included 11 body segments: arm (two), forearm (two), trunk (one), thigh (two), shank (two) and foot (2). (E) Increased cost of transport was evident following stroke. (F–H) External mechanical work (W_ext_) was greater for stroke survivors. Data are Mean±s.d., two-way ANOVAs (speed and lesion) followed by Tukey’s *post-hoc* test, *P<0.05 between groups as indicated, *n*_Stroke_=7, *n*_Control_=10 for C and *n*_Stroke_=6, *n*_Control_=10 for mechanical work variables; W_v_, vertical external mechanical work; W_f_, forward external mechanical work.

Post-stroke participants expended more metabolic energy than controls at 40% of their preferred walking speed (PWS) (speed: *F*_1,30_=4.602, *P*=0.041; lesion: *F*_1,30_=14.16, *P*=0.0007). The absence of posthoc effects at PWS (*P*>0.05) indicates that post-stroke participants were more economic at PWS than at 40% PWS, but not in comparison with controls, which is in line with the U-shape of C (Fig. 2E). Mechanical external work (W_ext_) is higher in post-stroke participants than control. W_ext_ production was increased for post-stroke participants at 40% PWS (*P*<0.05; speed: *F*_1,28_=13.10, *P*=0.001; lesion: *F*_1,28_=13.05, *P*=0.001; Fig. 2F). While most of the variation in the vertical component of W_ext_ was explained by lesion (32.29% of W_v_ variation; speed: *F*_1,28_=10.94, *P*=0.003; lesion: *F*_1,28_=18.62, *P*=0.0002; Fig. 2G), the main source of variation was speed (31.66% of W_f_ variation; speed: *F*_1,28_=16.57, *P*=0.0003; lesion: *F*_1,28_=7.775, *P*=0.009; Fig. 2H). The absence of posthoc effects at PWS (*P*>0.05) indicates that stroke survivors manage to reduce W_ext_ production by using a faster walking speed on the treadmill. This may reflect the need to walk faster in the forward direction, thus, stroke survivors may reduce the W_v_ to increase the horizontal velocity.

### W_seg_ production

The upper- and lower-body linear displacement in relation to BCoM are components of the internal energy of the system. To understand the contribution of upper- and lower-body segments to the increase in metabolic energy seen following a stroke, we implemented a new analysis based on the mechanical work production by each segment, i.e. shank, thigh, arm and forearm. The horizontal displacement of upper- and lower-body centers of mass (CoMs) in relation to BCoM (W_seg,f_) was greater for the non-paretic thigh and arm (interaction: *F*_14,152_=29.66, *P*<0.0001). The vertical displacement of body segments in relation to BCoM (W_seg,v_) was also greater for the non-paretic arm, but was also increased for the paretic thigh (interaction: *F*_14,152_=11.30, *P*<0.0001). Interestingly, the W_seg,f_ of the paretic thigh displayed a substantial correlation with the metabolic cost in stroke survivors at 40% PWS (*P*=0.066, r=0.777). Shank linear movements were reduced in the non-paretic side (*P*<0.05). Overall, these results reflect the asymmetries and compensations typically found in the hemiplegic gait, including the great reliance on the non-paretic side (Table 2).

### Pendular transduction within the step

**Fig. 3.**
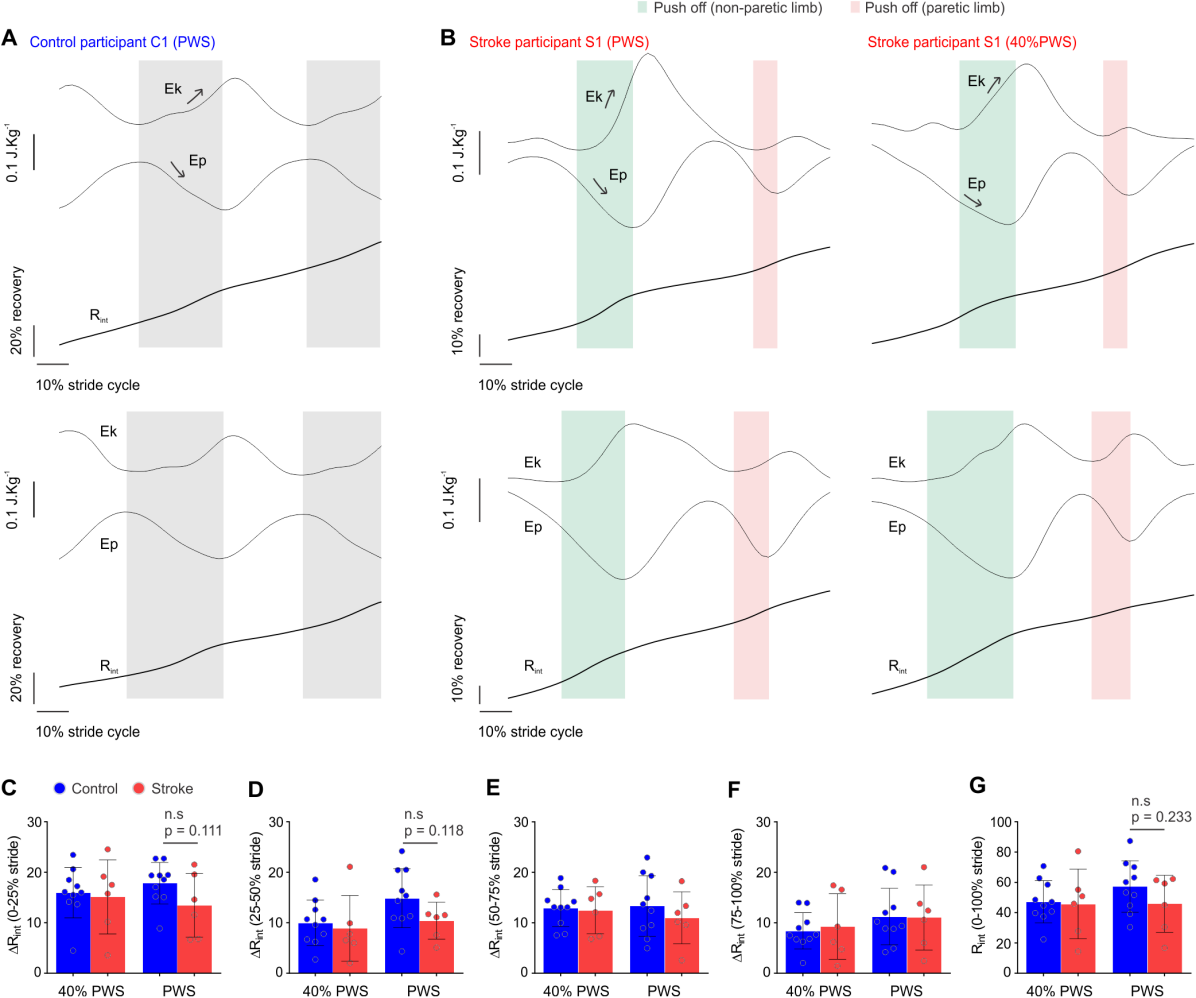
**Post-stroke gait displayed a substantial reduction of energy recovery within the stride.** (A) BCoM, Ep and Ek for a control participant, the stride cycle is defined as the time period of contact of the heel strike to next contact of the same limb; note the accumulation of energy recovery when exchanging Ep to Ek with both limbs (grey). (B) For post-stroke participants the stride cycle is defined as the time period from contact of paretic limb with the treadmill to the next contact of the same limb. During the initial contact of the paretic limb with the treadmill, stroke survivors used the paretic limb as a rigid shaft while pushing off using the non-paretic lower limb (green); but a substantial reduction of energy recovery occurred when exchanging Ep to Ek using the paretic limb to push off (red). Note that this transduction seems to occur more briefly and with a reduced increase in Ek. (C–F) Stroke survivors showed relative maintenance of energy recovery while walking at 40% of PWS. A substantial reduction of the energy transduction within the step (ΔR_int_) accumulated from 0–25%, 25–50% and 50–75% of the stride was evident for stroke survivors walking at the PWS. (G) R_int_ during the full stride was substantially reduced for stroke participants at the PWS; note that stroke participants maintain a relatively constant energy recovery, regardless of speed, whereas the control group shows the expected increase in energy recovery at PWS. Data are Mean±s.d.; two-way ANOVA (speed and lesion) followed by Tukey’s *post-hoc* test, *P<0.05 between groups as indicated, *n*_Stroke_=6, *n*_Control_=10. R_int_, energy transduction within the step.

At 40% PWS, post-stroke subjects displayed maintained energy recovery during the double support and single support phases while contacting the ground with the paretic limb, using it as a rigid segment, and forward pushing using the non-paretic limb to exchange Ep into Ek (Fig. 3B). This strategy does not hold for the PWS: although not significant, stroke survivors showed a reduction of R_int_ when the support foot is that of the paretic side (0–25% and 25–50% of the stride; *P*=0.111 and *P*=0.118, respectively; Fig. 3C,D). When using the paretic limb to push forward during the double support phase, energy conversion is also slightly reduced (Fig. 3E,F) and takes over during a reduced period (Fig. 3B). R_int_ accumulated during the full stride was substantially lower for post-stroke participants at PWS (R_int_ 0–100% stride: control=57.2%±16.9%, post-stroke=45.9%±18.9%; *P*=0.233; Fig. 3G).

## DISCUSSION

This study aimed at investigating the metabolic cost and conservation of energy in stroke survivors. Here, we corroborated the greater metabolic and mechanical demands of post-stroke gait and provided a detailed report on how the upper- and lower-limbs contributed by increasing the internal energy of the system. The most striking findings of this analysis depict how this greater internal energy was largely related to exaggerated movements between BCoM and upper- and lower-body non-paretic segments. Interestingly, the paretic lower-limb also contributed by increasing vertical internal energy. Compensation occurred in the non-paretic forearm, where an increase in forearm rotation was accompanied by greater vertical and horizontal arm movements. The post-stroke motor system also adapted to walking speed by reducing C and W_ext_, an effect expected when transitioning from speeds below the PWS to the PWS. Conversely, the energy conservation through pendular mechanics did not increase with speed and was reduced when the supporting foot was that of the paretic side; likely indicating reliance on the least-affected side at more challenging speeds. Overall, these chronic gait adaptations reflect how the neural system adapts to the loss of supraspinal drive in maintaining gait efficiency at the system level.

It is a consensus that increased work, not decreased efficiency, explains the greater metabolic cost of hemiplegic gait (Farris et al., 2015; Stoquart et al., 2012). The nervous system adapts to save energy (Selinger et al., 2019), for example, the transition to bipedal locomotion was one of the most remarkable features of human evolution (Lieberman, 2015). By adopting the bipedal posture, humans were able to save metabolic energy by optimizing pendulum and spring-mass mechanics (Saibene and Minetti, 2003). Post-stroke, these mechanisms of energy optimization are not as effective (Detrembleur et al., 2003). For example, here, post-stroke participants expended more metabolic energy than controls at speeds slower than PWS, following the twofold increase in C after stroke (Detrembleur et al., 2003; Stoquart et al., 2012). From a systems biology perspective, the locomotion pattern seen following stroke reflects the mechanical adjustments in attempting to minimize the cost while maintaining a functional gait, in spite of the higher mechanical work. Nevertheless, psychological constraints may induce patients to choose slower speeds of walking due to fear of falling, motor coordination impairment, reduced endurance and poor balance (Drużbicki et al., 2016). Here, walking on a treadmill using the PWS measured during overground walking yielded similar mechanical and metabolic outcomes to controls, and dissimilarities were shown at 40% PWS. The fact that post-stroke participants were less economic at a speed below the PWS may indicate maladaptive speed selection when choosing ‘safer’, slower speeds on the treadmill (U-shaped curve of C) (Saibene and Minetti, 2003). In accordance with our findings, stroke survivors also display greater walking symmetry while walking at faster speeds (Awad et al., 2015), and may change their gait symmetry to walk faster with a lower C (Roemmich et al., 2019). Thus, our results are under previous findings of reduced energy consumption at PWS, but also suggest that stroke survivors were able to maintain this U-shaped pattern. Indeed, as aforementioned, here we show how a speed below the PWS was metabolically and mechanically demanding, especially for stroke survivors. At the faster speed, one possible explanation may be that walking on a treadmill may induce greater symmetry between paretic and non-paretic hemibodies due to the restricted belt moving at a constant speed (Stoquart et al., 2012). To understand what factors contributed to this changing metabolic demand at different speeds, we also investigated the mechanical adaptations encompassing BCoM and CoM interactions.

Mechanical work to accelerate BCoM in relation to gravity and the environment during walking (i.e. W_ext_) and to move segments in relation to BCoM (i.e. W_int_) have a well-consolidated relation with the metabolic demands of walking (Saibene and Minetti, 2003; Stoquart et al., 2012). A few studies have documented this increase of W_ext_ in stroke survivors (Detrembleur et al., 2003; Fábrica et al., 2019; Mahon et al., 2015; Stoquart et al., 2012). Here, we corroborate these findings of increased W_ext_, which was mainly related to W_v_ production. Here, also, the use of kinematics furthered a more detailed investigation focusing on internal energy production.

Thus, we conducted a segmental analysis of internal mechanical energies. This analysis allowed us to identify the asymmetrical nature of internal energy production following a stroke. This segmental analysis unveiled that the non-paretic upper limb showed increased compensation, evident in the increased forward and vertical arm movements, likely to stabilize the BCoM. This forearm kinetics increased the internal energy of the system. Some subjects may display exaggerated interlimb neural coupling following stroke, which leads to significant muscle activity in the arm during walking (Kline et al., 2007). Similarly, upper-body segments (e.g. arms, head and trunk) have been implicated in increasing the mechanical energy cost of the system following stroke (Olney et al., 1986). Further studies are needed to better understand how the wide range of post-stroke hemiplegic gait impairments may reflect the mechanical consequences of muscle weakness, spasticity, abnormal synergistic activation, and their interactions (Li et al., 2018).

We also found that the paretic lower limb transitioned with a greater vertical contribution to the internal energy of the system. This may reflect the hemiparetic leg impairment, often characterized by stiff knee, hip circumduction and plantarflexion, leading to compensatory movements to facilitate toe clearance (Kerrigan et al., 1999, 2000). This compensation was not reduced when adopting faster speeds, although C and W_ext_ were reduced. This is consistent with previous findings that related faster treadmill walking with a more normal walking pattern after stroke without changes in gait compensations, such as circumduction (Tyrell et al., 2011). The hemiplegic gait also displays shorter stride lengths with a prolonged swing phase on the paretic side (Fábrica et al., 2019). We can speculate that the reduced internal energy production within the non-paretic shank (vertical and horizontal) and thigh (vertical) may reflect the stabilizing role of the non-paretic limb to assist in this transition, keeping the non-paretic supporting leg in phase with the BCoM by using shorter steps. Overall, internal energy production following a stroke was characterized by non-paretic upper-limb compensation and also by vertical contributions of the paretic leg.

Finally, we reported R_int_ similar to controls during treadmill walking at 40% PWS. Post-stroke participants were likely able to optimize pendular exchange using the paretic limb as support and the non-paretic limb as an actuator during push-off. Paresis affects muscular activation and strength, and as such the use of the paretic leg as a more rigid and passive segment at the slowest speed may optimize the inverted pendulum mechanics with reduced muscular action (Bowden et al., 2006). The paretic lower limb displays a lower magnitude period of push-off (Farris et al., 2015), which, here, may have influenced energy recovery when the paretic limb was pushing the treadmill while the non-paretic limb was supporting the BCoM, especially at the faster speed. As such, the post-stroke gait lacked the marked characteristic of increasing recovery while transitioning from a speed below the PWS to PWS, evident in controls PWS (R_int_ 0–100% stride: 40% PWS≈47%; PWS≈57%) (Saibene and Minetti, 2003). Our energy recovery data (stroke≈45%, control≈57%) is in agreement with previously-reported data for level walking at PWS (stroke≈50%, control≈56%; Fábrica et al., 2019), but displayed high variability. Although studies conducted during treadmill (Minetti et al., 1995) or overground walking (Balbinot, 2017) reported similar recovery amplitudes, in the present study, a possible explanation is the emergence of anomalous BCoM energy fluctuations during treadmill use. Collet and collaborators showed how some participants may employ a low-recovery walking strategy while walking on the treadmill (Collett et al., 2007). This may have increased the variability in the recovery profiles and hampered our study in detecting a statistical significance for this parameter, although several trends appeared and corroborated previous findings of lower R_int_ for stroke survivors (Fábrica et al., 2019).

### Limitations and future work

There were some limitations in our study design, such as the CO_2_ production in the metabolic cost analysis, which may bias the C outcomes at faster speeds. Further work is needed to better understand the relationship between gait compensations and asymmetries with the internal energy production under the classical W_int_ production framework (Saibene and Minetti, 2003; Willems et al., 1995), including mechanical simulations of the post-stroke gait pattern. Also, we acknowledge the debate in the literature and current limitations to determining mechanical work.

Elevated cost of walking is a particular concern for chronic stroke survivors because it leads to decreased activity tolerance and consequently, a sedentary lifestyle. Rehabilitation strategies should consider not only the management of spasticity, range of motion maintenance and motor function, but also task-specific treadmill-gait training to equalize inter-segment coordination and reduce mechanical work (Selgrade et al., 2017). Our study found that walking on a treadmill at faster speeds may be advantageous for stroke survivors. We suggest that rehabilitation efforts to improve gait should not use speeds lower than the PWS on a treadmill. These findings should further studies on how speed may affect the post-stroke gait, especially when dealing with split-belt treadmills, which can add an interesting independent speed control over paretic and non-paretic limbs. Split-belt training has been recommended in correcting step-length asymmetries in stroke survivors (Reisman et al., 2013). Nevertheless, more studies are necessary to understand not only how the faster belt induces a longer swing phase (mimics a paretic limb), but also how much extra muscular work is produced during the contact phase of this same limb (a non-paretic limb demand) (Tesio et al., 2018). Locomotor adaptation of walking is thought to involve a reactive feedback control of stance time, not related to supraspinal control, but to central pattern generators in the spinal cord (Lam et al., 2006; Reisman et al., 2005). In line with this, split-belt training may reduce the non-paretic/paretic asymmetry in stance by reactive feedback and, thus, without pronounced reliance on upper motor neuron control. This may be of particular importance when developing rehabilitation interventions for stroke patients using different split-belt speed ratios (Yokoyama et al., 2018).

### Conclusions

At the systems level, the findings described here reflect the complex neural adaptations of the locomotor system seen following a stroke. The most striking findings of this study unveil the asymmetrical nature of internal energy production and the speed-dependent C and W_ext_ during treadmill walking. Walking activities are often employed in post-stroke rehabilitation, both in acute and chronic phases of recovery, and treadmill walking is one of the most accessible activities and interventions. Improvements in post-stroke walking function may be achieved through treadmill gait training (Awad et al., 2015, 2016; Pohl et al., 2002), which may improve spatiotemporal gait asymmetry and reduce the energy cost of walking following stroke. Overall, our findings further the development of novel rehabilitation treatments focused on gait mechanics and energetics, for example, by using visual biofeedback to achieve improved coordination between segments or when using assistive devices (Bae et al., 2018). In this line of thought, previous studies showed that reductions of BCoM vertical displacement (of ≈10%) using real-time feedback may save 30% of the energy cost of locomotion (Massaad et al., 2010). Similar approaches may be used to reduce internal energy production, focusing on CoM and BCoM, with potential impact over the metabolic cost of locomotion.

## MATERIALS AND METHODS

### Participants

Stroke survivors were recruited from local stroke support groups and clinics. Participants without disabilities were recruited from local university communities. All participants were screened according to the following inclusion criteria, as described elsewhere (Mahon et al., 2015): (1) age range between 40–80 years, (2) no orthopedic surgeries within 6 months of the study, (3) obtained ‘minimally active’ score for level of physical activity evaluated by International Physical Activity Questionnaire (IPAQ) (Helmerhorst et al., 2012). Additional inclusion criteria for the stroke-group participants were that they (1) were at least 6 months post stroke, (2) had only experienced one stroke, (3) were capable of ambulation without use of an assistive device or orthosis, (4) were able to walk without assistance on a treadmill over sufficient time to complete the metabolic analysis, (5) were able to ambulate at velocities above and below PWS; (6) were not currently receiving lower extremity botulinum toxin injections, (7) were medically stable, (8) had no or only slight spasticity in their arms and legs (Ashworth scores 0 and 1). Control participants were additionally screened according to the following inclusion criteria: (1) they were able to walk independently on a treadmill over sufficient time to complete the metabolic analysis and (2) they had no known neurological condition or deficits.

Sample size was based in a previous study (Detrembleur et al., 2003). This study involved seven chronic stroke patients and ten healthy age-matched subjects (Table 1). One stroke participant was excluded from the kinematics analysis due to poor video quality. All participants signed written informed consent forms and the research protocol was approved by the Ethics Committee in human studies (n. 17434).

**Table 1.**
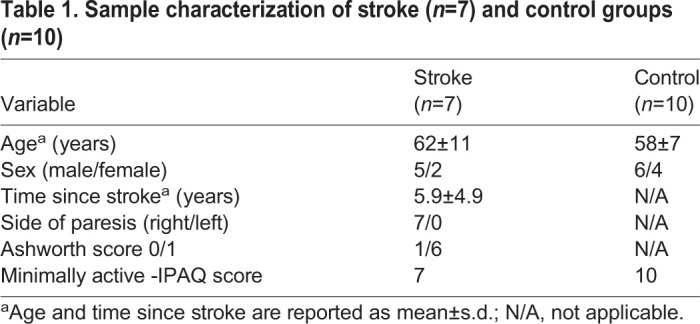
**Sample characterization of stroke (*n*=7) and control groups (*n*=10)**

**Table 2.**
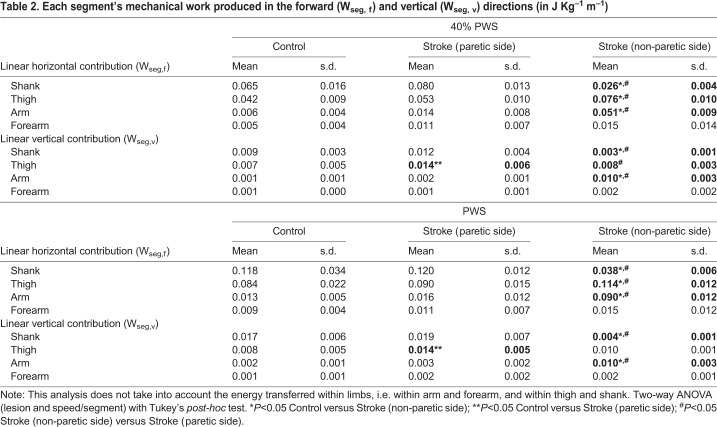
**Each segment's mechanical work produced in the forward (W_seg, f_) and vertical (W_seg, v_) directions (in J Kg^–1^ m^–1^)**

### 3D kinematics and metabolic instrumentation

Bilateral kinematic data were acquired using a four-camera system (JVC GR-DVL 9800, sample rate of 50 Hz for 60 s, JVC Company of America, Wayne, NJ, USA) positioned on both sides of the treadmill (Explorer Pro Action, BH Fitness, Vitoria-Gasteiz, Àvala, Spain). We calibrated the treadmill velocity by digitizing an adhesive retro-reflective marker on the tread belt as it traveled along the length of the treadmill. Optimum treadmill location concerning positioning of all cameras was set to ensure that all spatial coordinates were referenced to a single laboratory coordinate system. Oxygen consumption (VO_2_) metabolic system (VO2000, Medgraphics, St Paul, MN, USA) and heart rate monitor were used to provide breath-by-breath gas analysis and heart rate, respectively (Fig. 2C).

### Familiarization and testing

A pre-test day was designated as a familiarization session to ensure habituation to treadmill walking at different speeds. Based on studies that assess pathological locomotion, there is a limitation of progression of speed in treadmill walking for hemiparetic subjects ranging from 1.7 to 3.6 km h^−1^ (Brouwer et al., 2009; Detrembleur et al., 2003). Thus, walking speeds were encompassed approximately in this range. In the familiarization session, the PWS was measured during level walking over a 10 m path (Fig. 2A). Two or three speeds below the PWS were used, typically ranging from 22% to 66% of the PWS. One to two speeds slightly above or below the PWS were also clustered, and typically ranged from 85% to 114% of the PWS. These speeds were clustered in two groups: control 40% PWS (mean speed 42.60%±16.28% PWS), control PWS (mean speed 100.64%±15.10% PWS), stroke 40% PWS (mean speed 45.24%±9.88% PWS) and stroke PWS (mean speed 97.92%±12.84% PWS. Speed values in km h^−1^ are shown in Fig. 2B. On the test day, 18 reflective markers (15 mm diameter) were attached bilaterally to landmarks that deﬁned segment extremities (immediately anterior to tragus of ear, shoulder, lateral epicondyle of humerus, ulnar styloid process, greater trochanter and lateral epicondyle of femur, lateral malleolus, calcaneus and fifth metatarsal head) (Mian et al., 2006; Minetti et al., 1993). From the 3D positions of the 18 anatomical markers, we built a spatial model of 11 rigid segments: head–neck–trunk, upper arms, lower arms, thighs, lower legs and feet (Gomeñuka et al., 2014; Minetti et al., 1993). This spatial model enabled the calculation of the segments’ centers of mass and segment length required to build the kinematic model (head–trunk, forearms, arms, thighs, shanks and feet) (Fig. 2D).

After reflective markers positioning, subjects remained in a standing position for 5–7 min for measurement of their resting metabolic rate. Next, as aforementioned, participants walked at four or five different walking speeds on the treadmill, randomly. The subjects walked for 5 min at each speed. All kinematic and metabolic measurements were recorded over the last minute of walking at each speed. Heart rate and oxygen consumption were also measured between each trial, and this information was used to control when to start a new trial, i.e. to allow subjects to return to basal metabolic conditions.

### Data processing and analysis

The gait event detection was performed by visual inspection of the video images, and the stride was defined from paretic limb touch down to paretic lift off (Gomeñuka et al., 2014). VO_2_ was used to calculate C. Briefly, the VO_2_ at rest was subtracted from VO_2_ during the fourth and fifth minutes of the treadmill walking trial, divided per total body mass and distance traveled (in meters) (Farris et al., 2015; Margaria, 1938). C was expressed in joules per kilogram and meter (J kg^−1^ m^−1^). Metabolic data were converted to joules using an energetic equivalent of 20.1 J (ml O_2_)^−1^ (Detrembleur et al., 2003). Since the metabolic cart did not allow us to collect CO_2_ we used a fixed respiratory exchange ratio value.

Mechanical work and energy recovery data were calculated using 3D kinematic modeling and analyzed as described elsewhere (Balbinot, 2017; Cavagna et al., 2002; Minetti et al., 1993; Willems et al., 1995). Data were filtered by a low-pass, fourth-order Butterworth filter at a cutoff frequency determined by Winter's residual analysis (Winter, 2009). Anthropometric tables data of 11 rigid segments were used to compute the CoM and BCoM positions (Pavol et al., 2002). Linear and angular velocity of each CoM and linear velocity of BCoM were calculated using mathematical derivatives. Computational algorithms were constructed to calculate mechanical work using LabVIEW^®^ 8.5 (National Instruments, Austin, TX, USA).

BCoM potential (*E_p_*), kinetic (*E_k_*) and total (*E_tot_*) energies were calculated as follows:(1)
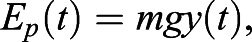
(2)
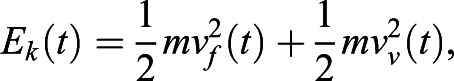
(3)
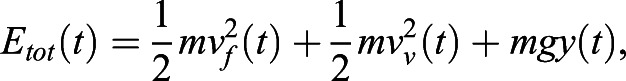
where *m* is body mass in kg, *v_f_*(*t*) is the forward velocity of BCoM in m s^−1^, *v_v_*(*t*) is the vertical velocity of BCoM in m s^−1^, *g* is acceleration of gravity (9.81 m s^−2^) and *y* is the vertical position of BCoM.

BCoM velocities were calculated using the first derivative of horizontal [*v_f_*(*t*)] and vertical [*v_v_*(*t*)] position of the BCoM. *E_p_* and *E_k_* energies were used to calculate forward (*W_f_*) and vertical (*W_v_*) external mechanical work, as follows (Willems et al., 1995):(4)
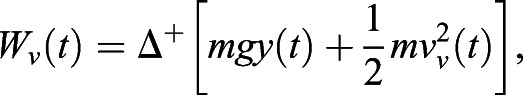
(5)
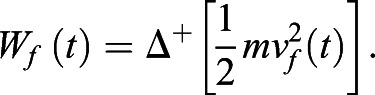


*W_ext_*, work required to move the BCoM in relation to the environment, is the sum of positive increments of *E_tot_*. *W_ext_* is given by Cavagna and Kaneko, 1977; Cavagna et al., 1976 and Minetti et al., 1993 as:(6)
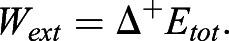
CoM linear kinetic energies were used to calculate each segment's work, the linear horizontal contribution (*W_seg_*_,*f*_) and vertical contribution (*W_seg_*_,*v*_). This novel method of calculation sought to explore the extra mechanical work caused by abnormal compensations and asymmetries seen following a stroke, as follows:(7)
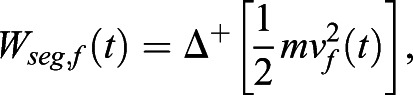
(8)
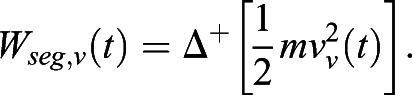


The above-mentioned analysis, Eqns 7 and 8, differs from the classical internal mechanical work (*W_int_*) calculation (Saibene and Minetti, 2003; Willems et al., 1995). It stems from the idea that asymmetrical displacement of segments may cause the movement of BCoM, due to the lack of counterbalanced (opposite) movement of another equivalent segment often seen in the paretic limbs of stroke survivors. Importantly, this analysis does not take into account the energy transferred within limbs, i.e. within arm and forearm, and within thigh and shank.

Interchanges between mechanical energies of BCoM within the step (pendulum-like mechanism) were quantiﬁed using *R*_*int*_ calculations (Cavagna et al., 2002), defined as:(9)
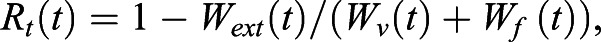
where *R* is the pendular energy transduction within the step, *t* is time, *W_v_* is the vertical external mechanical work, *W_f_* is the horizontal external mechanical work, and *W_ext_* is the external mechanical work.(10)
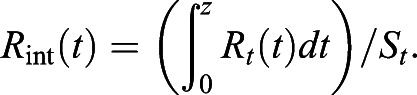
where *R*_*int*_ is the cumulative energy transduction within the step (%) and *S*_*t*_ is the stride time (s), (0 to z) is the integration period, and dt is 1/sampling frequency.

### Statistical analysis

All variables were tested for normality using the Shapiro–Wilk test. Two-way ANOVA was carried out to test for differences between groups (lesion and speed). For the analysis of each segment's work production, factors were lesion and segment/speed, and the lesion factor included three levels (control, stroke paretic and stroke non-paretic). When appropriate, analyses were followed by Tukey’s *post-hoc* test. The Pearson correlation was used to test the relation between C and the mechanical variables. For each participant, mechanical variables reported were determined over 15±4 (mean±s.d.) gait cycles at each speed. Values are mean±s.d. Statistical significance was set at *P*<0.05. Statistics were performed using GraphPad Prism software.
